# Supplementary material for: Conduction band convergence and local structure distortion for superior thermoelectric performance of GaSb-doped n-type PbSe thermoelectrics
Source: Nat Commun. 2025 Jul 1;16:5749. doi: 10.1038/s41467-025-60571-1 (PMC12216291; doi:10.1038/s41467-025-60571-1)
Supplement: Supplementary file 1 — Supplementary Information [file 41467_2025_60571_MOESM1_ESM.pdf]

Supporting Information

Conduction band convergence and local structure distortion for superior thermoelectric performance of GaSb-doped n-type PbSe thermoelectrics

Jing Zhou,[1,2,‡] Hong-Hua Cui,[3,‡] Yukun Liu,[4,‡] Hongwei Ming,[2] Yan Yu,[1,2] Vinayak P. Dravid,[4] Zhong-Zhen Luo,[1,2,5,*] Qingyu Yan,[6,*] Zhigang Zou,[1,2,7,8] Mercouri G. Kanatzidis[4,9,*]

[1]Key Laboratory of Advanced Materials Technologies, International (HongKong Macao and Taiwan) Joint Laboratory on Advanced Materials Technologies, College of Materials Science and Engineering, Fuzhou University, Fuzhou, 350108, P. R. China

[2]Fujian Science & Technology Innovation Laboratory for Optoelectronic Information of China, Fuzhou, Fujian 350108, China

[3]Mechanical and Electrical Engineering Practice Center, Fuzhou University, Fuzhou, 350108, China

[4]Department of Materials Science and Engineering, Northwestern University, Evanston, Illinois 60208, United States

[5]State Key Laboratory of Photocatalysis on Energy and Environment, Fuzhou University, Fuzhou 350116, China

[6]School of Materials Science and Engineering, Nanyang Technological University, 50 Nanyang Avenue 639798, Singapore

[7]Eco-materials and Renewable Energy Research Center, College of Engineering and Applied Sciences, Nanjing University, Nanjing, 210093, China

[8]National Laboratory of Solid State Microstructures, Nanjing University, Nanjing 210093, China.

[9]Department of Chemistry, Northwestern University, Evanston, Illinois 60208, United States

[‡]These authors contributed equally: Jing Zhou, Hong-Hua Cui, Yukun Liu

Corresponding authors: E-mail: zzluo@fzu.edu.cn (Z. Z. Luo),
E-mail: alexyan@ntu.edu.sg (Q. Yan),
E-mail: m-kanatzidis@northwestern.edu (M. G. Kanatzidis)

Single parabolic band (SPB) model

Generally speaking, the heavier density of the state effective mass m^* favors a larger *S*. In order to further understand the improvement in *S*, we calculated m^* using the single parabolic band (SPB) model.[1] According to the Boltzmann transport theory, m^* can be calculated by the following equations:

The Seebeck coefficient:

$$S(\eta) = \frac{k_B}{e}\left[\frac{(r+\frac{5}{2})F_{(r+\frac{3}{2})}(\eta)}{(r+\frac{3}{2})F_{(r+\frac{1}{2})}(\eta)} - \eta\right] \tag{S1}$$

The Hall carrier concentration:

$$n_H = \frac{1}{eR_H}\frac{(2m^*k_BT)^{3/2}}{3\pi^2h^3}\frac{(r+\frac{3}{2})^2F^2_{(r+\frac{1}{2})}(\eta)}{(2r+\frac{3}{2})^2F_{(2r+\frac{1}{2})}(\eta)} \tag{S2}$$

Here, $F_{(j)}(\eta) = \int_0^\infty \frac{\xi / d\xi}{1+\exp(\xi+\eta)}$ is the Fermi integral, k_B is the Boltzmann constant, η is the reduced Fermi level, and r = -1/2 when charge carriers are scattered by acoustic phonons, *h* is the reduced Planck constant, and $m^* = N_v^{2/3}m_b$, m_b is the band effective mass and N_v is the degeneracy of the band valleys.

L is Lorenz number, respectively. *L* was calculated according to the equation:

$$L = (\frac{k_B}{e})^2\left[\frac{3F_0(\eta)F_2(\eta)-4F_1(\eta)^2}{F_0(\eta)^2}\right] \tag{S3}$$

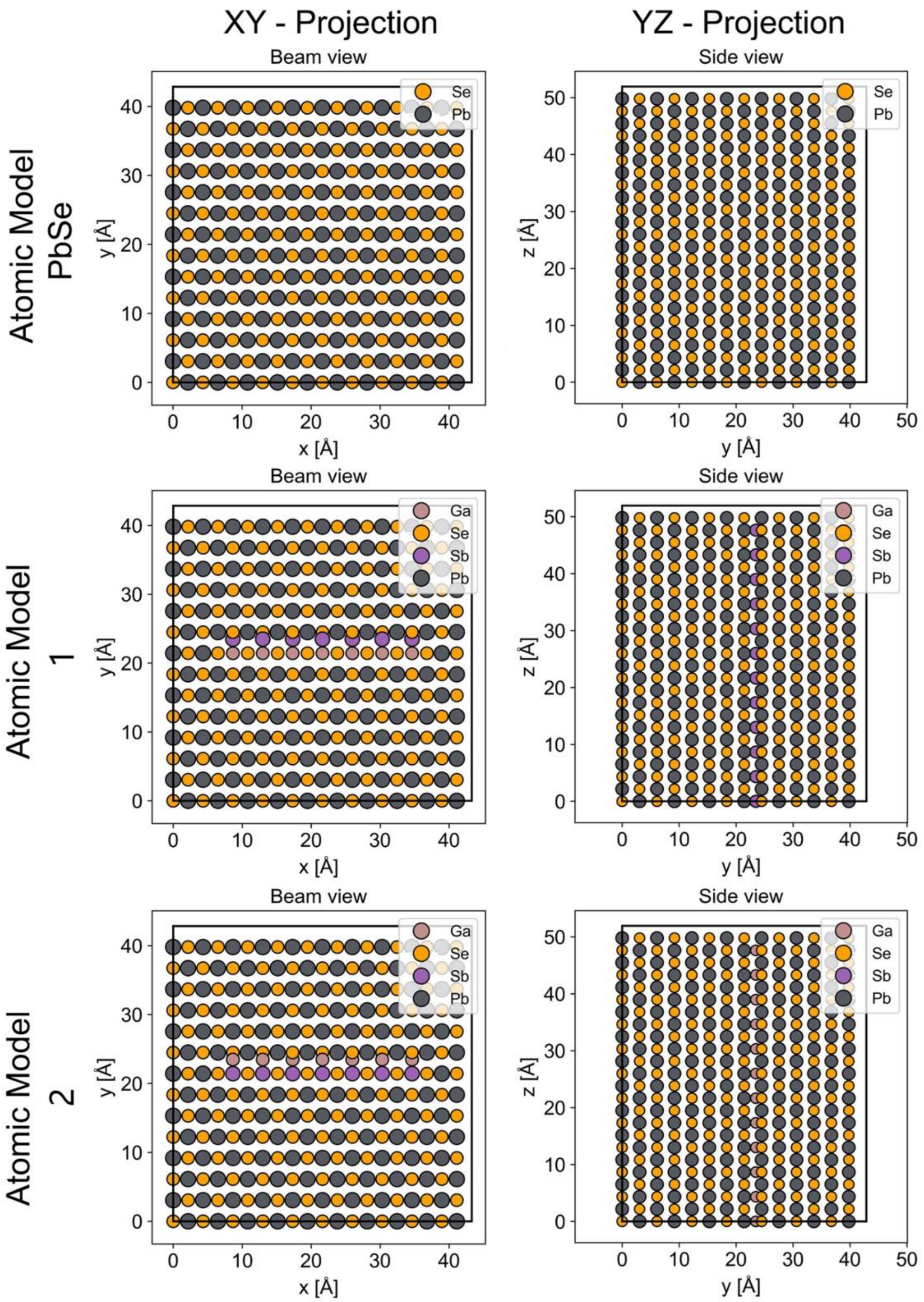

Figure S1. Atomic model for PbSe and $Pb_{1-x}(GaSb)_xSe$ with different configurations used for multislice simulation of HAADF images as shown in Figure S2 and Figure S3.

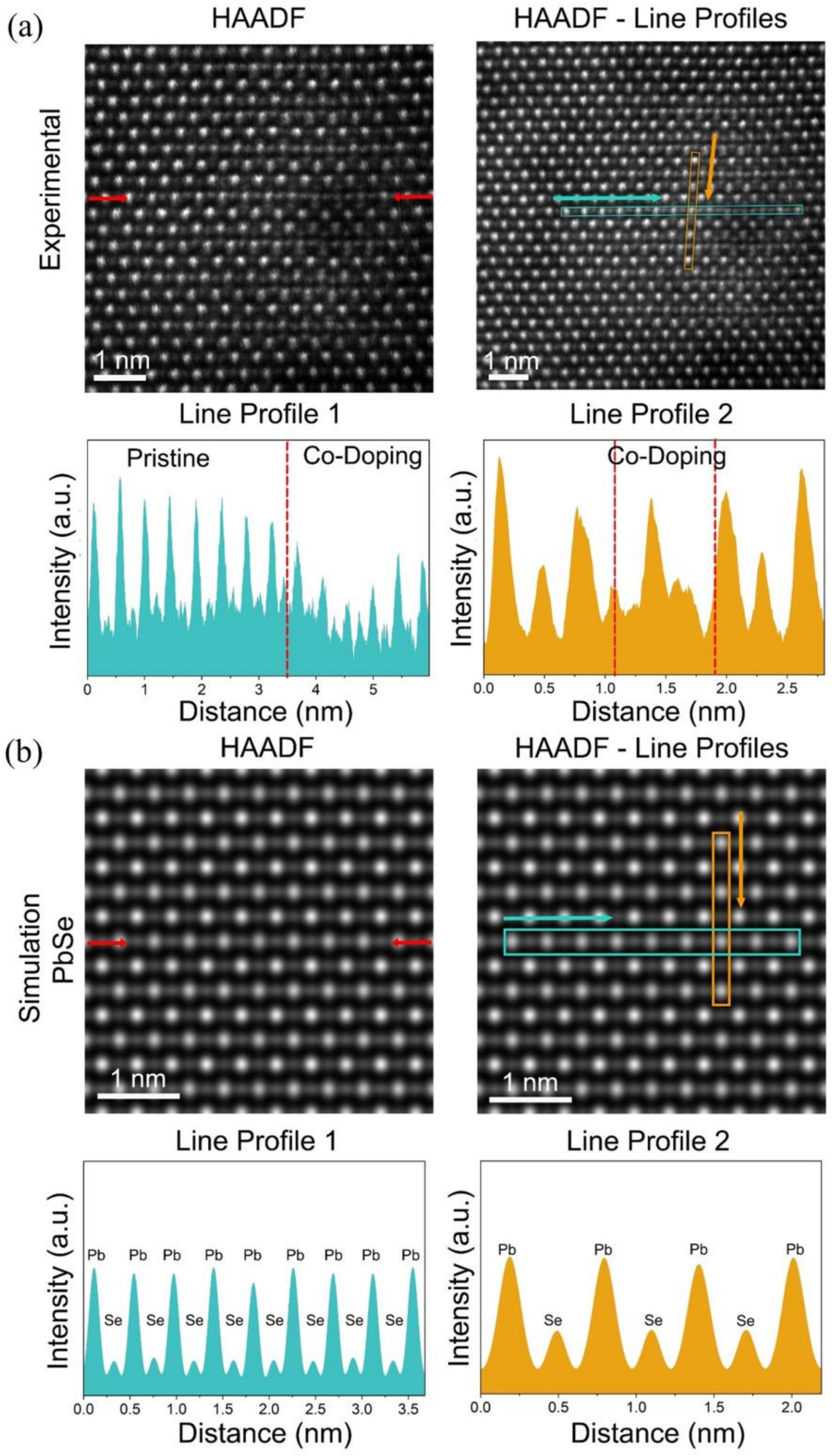

Figure S2. (a) Experimental atomic resolution HAADF image of $Pb_{0.99875}(GaSb)_{0.00125}Se$ and (b) multislice simulation of pristine PbSe. The atomic model used for simulation is shown in Figure S1.

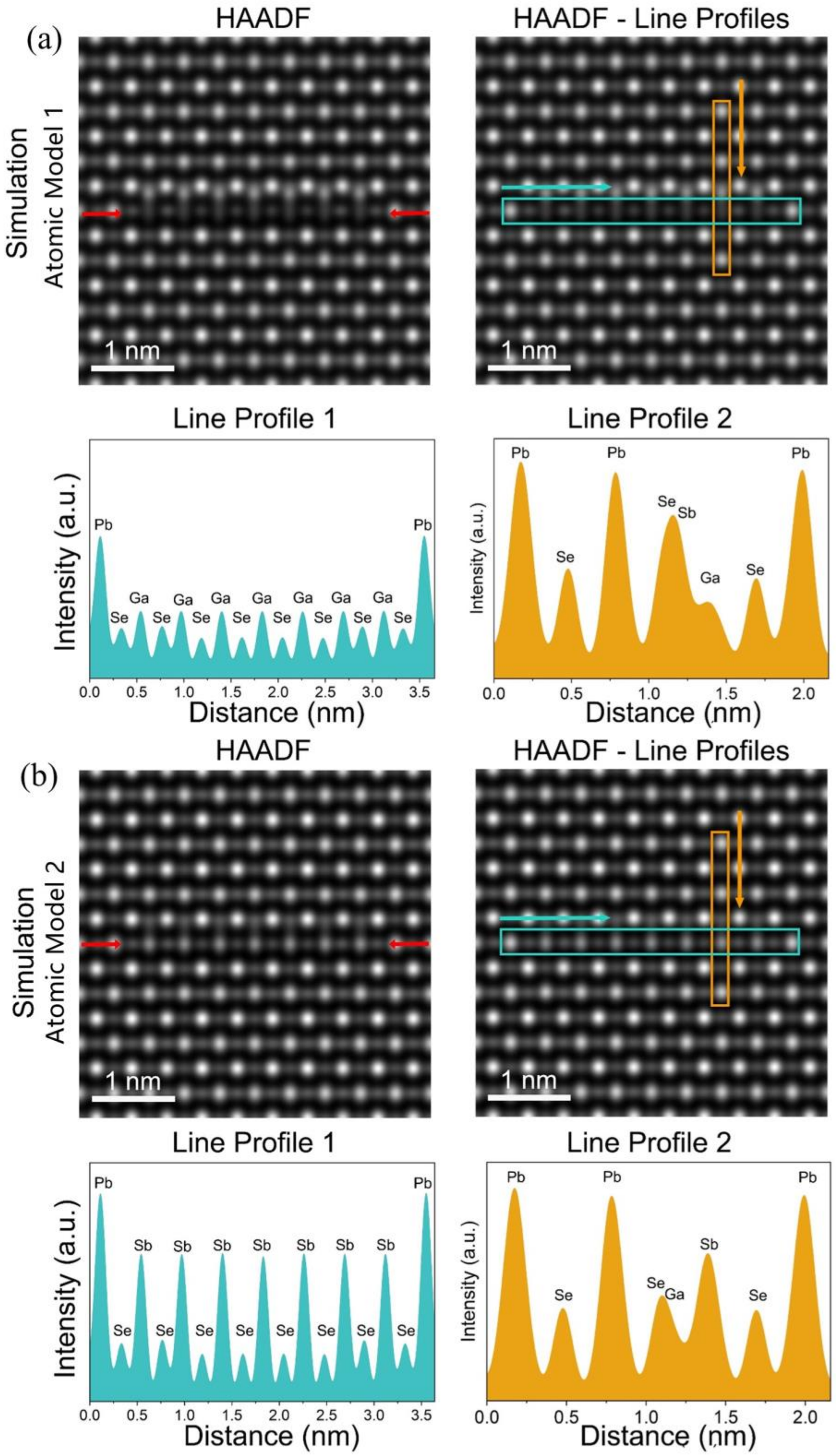

Figure S3. Multislice simulation of GaSb co-occupation with (a) Ga atom residing at the Pb site, and (b) Sb atom residing at the Pb site. The atomic models used for simulation are shown in Figure S1.

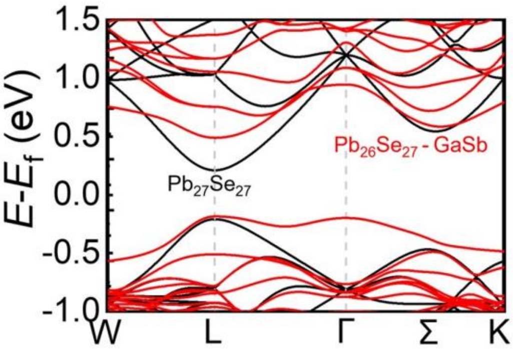

Figure S4. Electronic band structures for intrinsic PbSe and GaSb-doped PbSe ($Pb_{26}GaSbSe_{27}$).

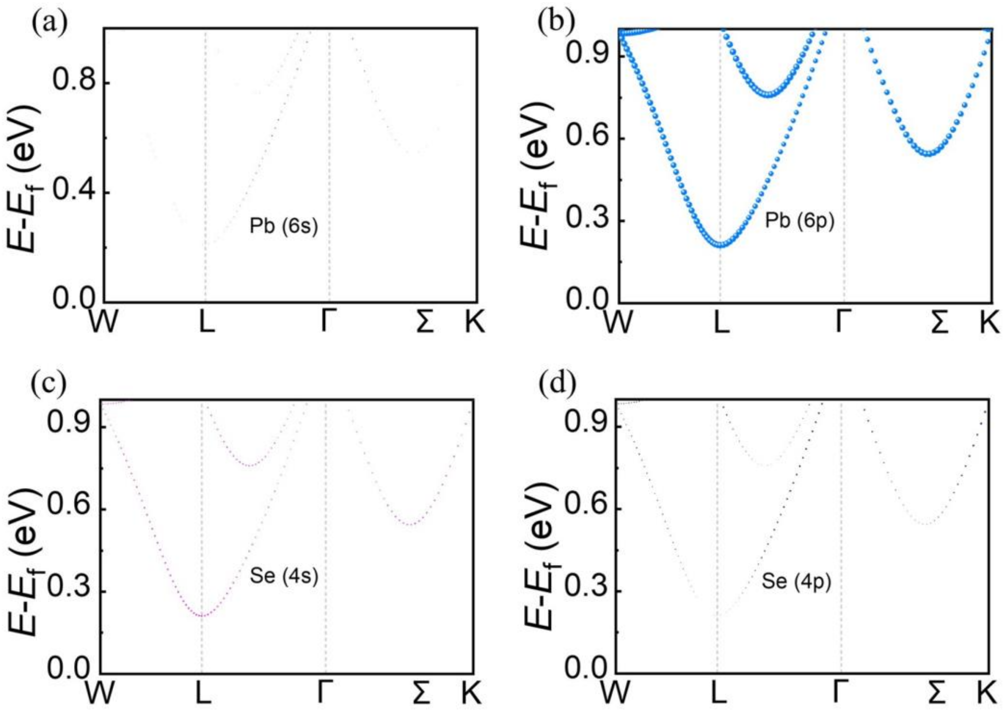

Figure S5. Contribution of Pb 6s (a), Pb 6p (b), Se 4s (c), and Se 4p (d) to the conduction structure of pure PbSe.

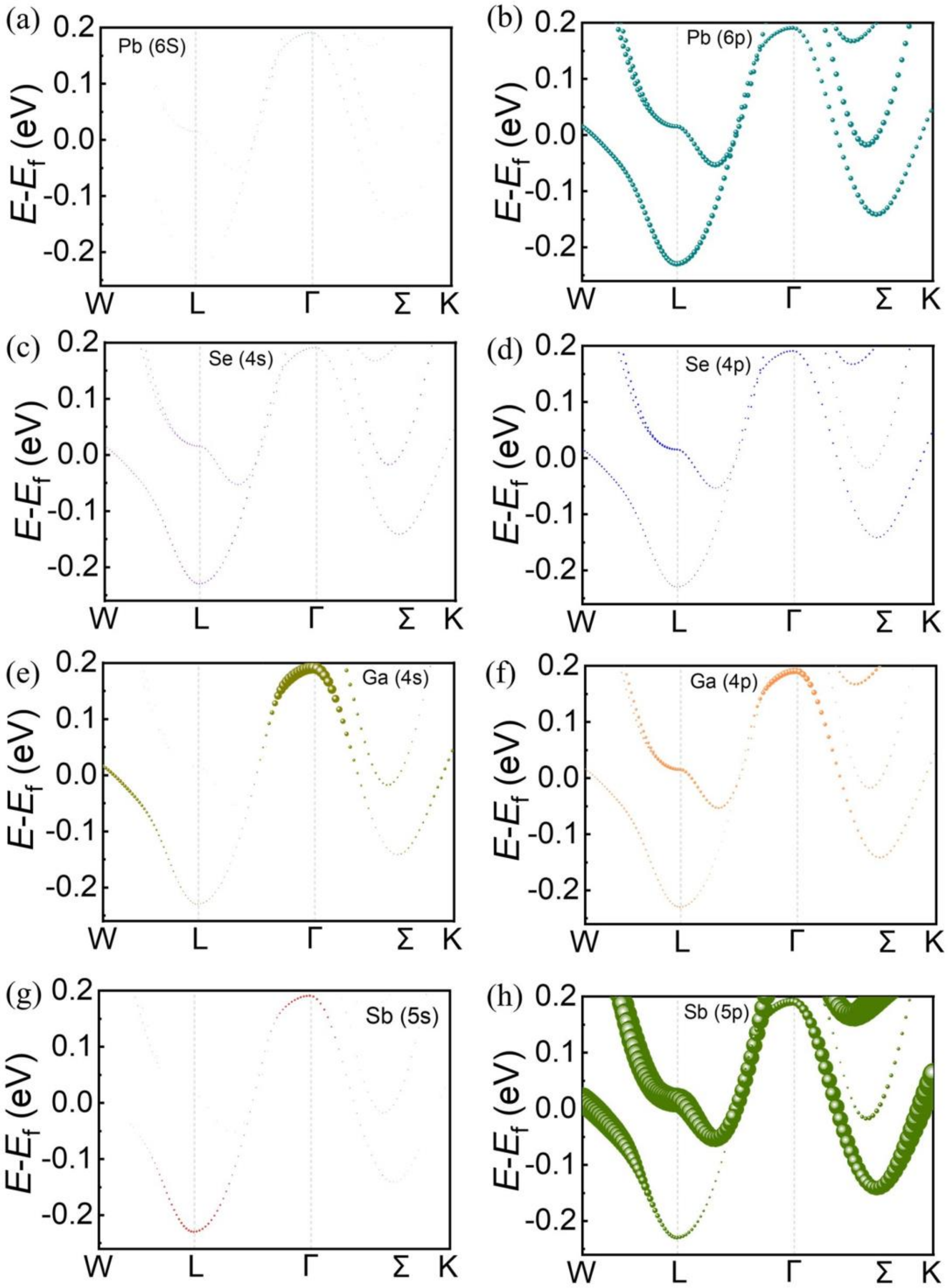

Figure S6. Contribution of Pb 6s (a), Pb 6p (b), Se 4s (c), Se 4p (d), Ga 4s (e), Ga 4p (f), Sb 5s (g), and Sb 5p (h) to conduction structure of GaSb-doped PbSe ($Pb_{26}GaSbSe_{27}$).

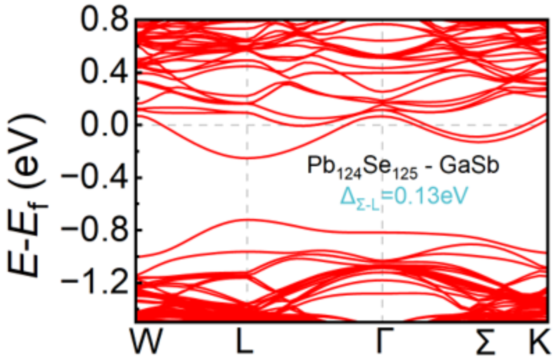

Figure S7. Electronic band structures of GaSb-doped PbSe ($Pb_{124}GaSbSe_{125}$).

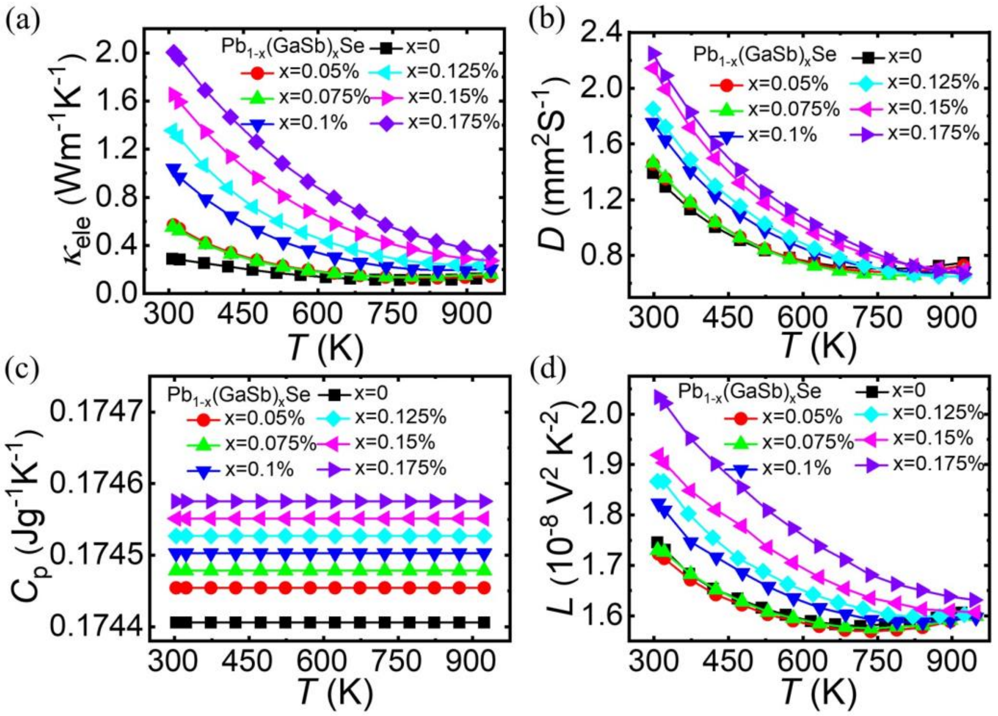

Figure S8. Temperature-dependent (a) electronic thermal conductivity, κ_{ele}; (b) thermal diffusivity, D; (c) heat capacity, C_p; and (d) Lorenz numbers, L for $Pb_{1-x}(GaSb)_xSe$ samples (x = 0, 0.05%, 0.075%, 0.1%, 0.125%, 0.15%, and 0.175%).

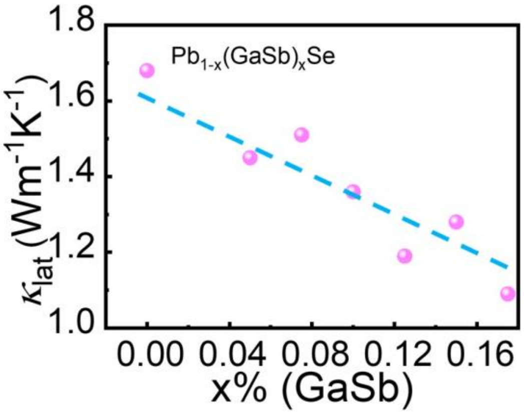

Figure S9. Lattice thermal conductivity, κ_{lat} of $Pb_{1-x}(GaSb)_xSe$ (x = 0, 0.05%, 0.075%, 0.1%, 0.125%, 0.15%, and 0.175%) as a function of GaSb semiconductor content at 300 K.

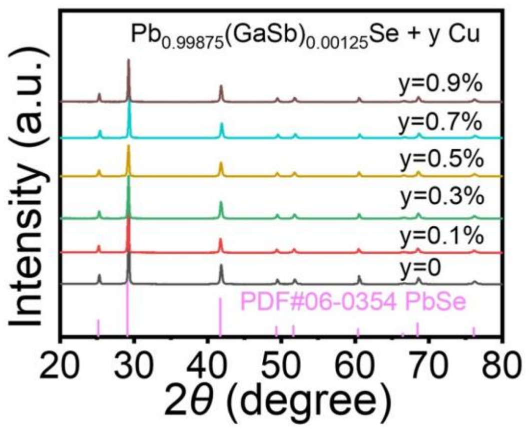

Figure S10. PXRD patterns of $Pb_{0.99875}(GaSb)_{0.00125}Se$-yCu (y = 0, 0.1%, 0.3%, 0.5%, 0.7%, and 0.9%) samples.

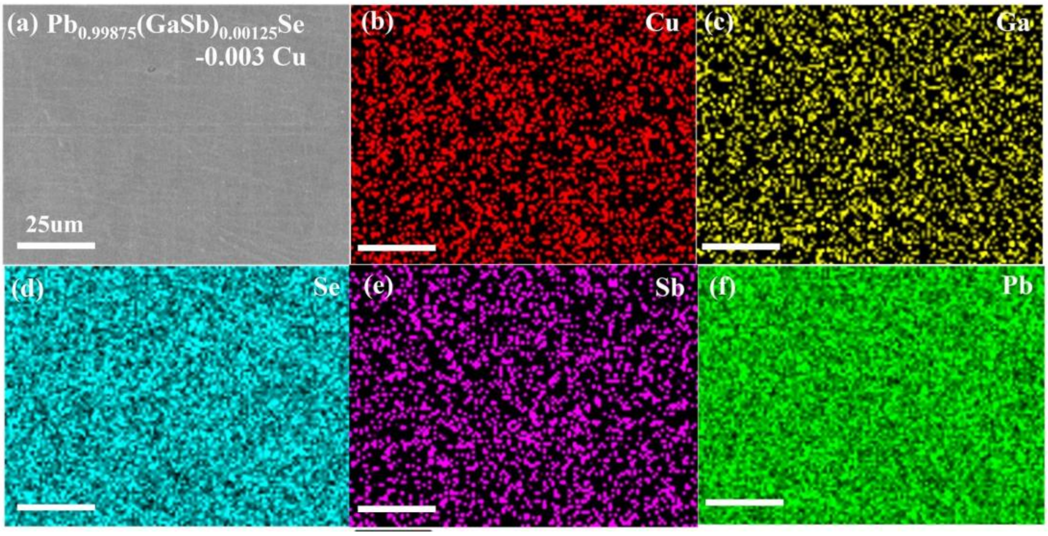

Figure S11. Microstructure and composition analysis of the $Pb_{0.99875}(GaSb)_{0.00125}Se$-0.3%Cu sample. (a) Back-scattered SEM (BSE) image of the specimen. (b-f) EDS mapping of the Figure S11 (a) region.

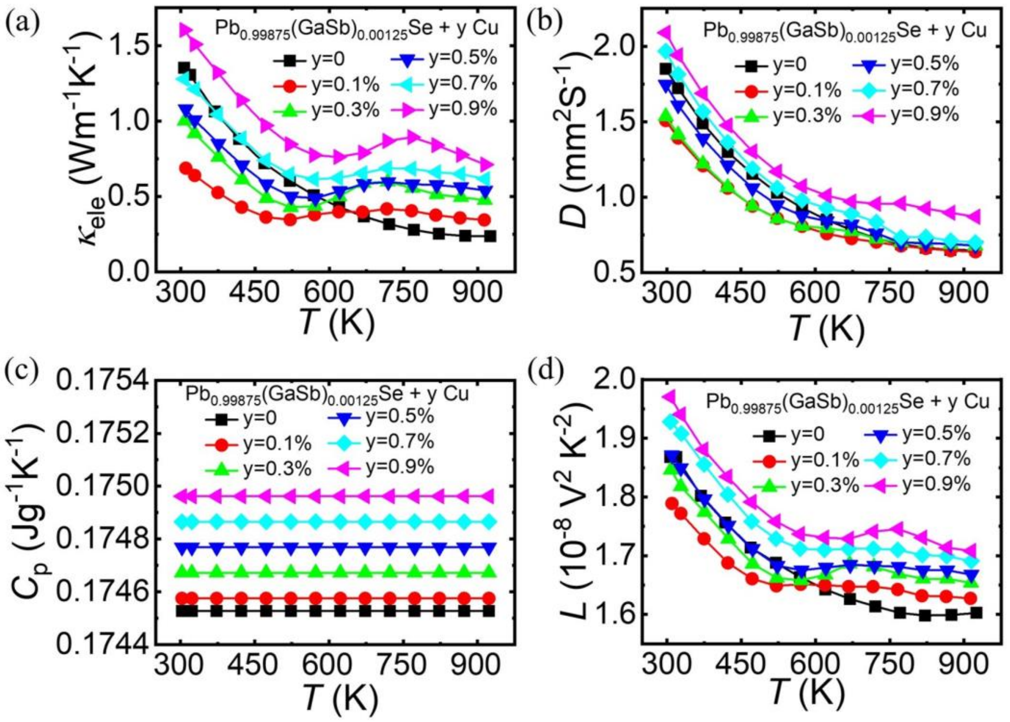

Figure S12. Temperature-dependent (a) electronic thermal conductivity, κ_{ele}; (b) thermal diffusivity, D; (c) heat capacity, C_p; and (d) Lorenz numbers, L for $Pb_{0.99875}(GaSb)_{0.00125}Se$-yCu (y = 0, 0.1%, 0.3%, 0.5%, 0.7%, and 0.9%) samples.

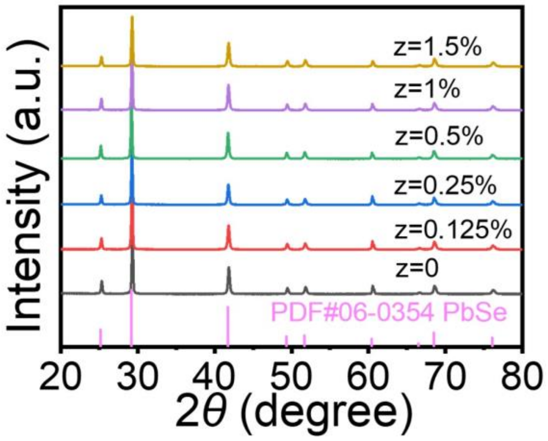

Figure S13. PXRD patterns of $Pb_{0.99875}(GaSb)_{0.00125}Zn_zSe_{1+z}$-0.3%Cu (z = 0, 0.125%, 0.25%, 0.5%, 1%, and 1.5%) samples.

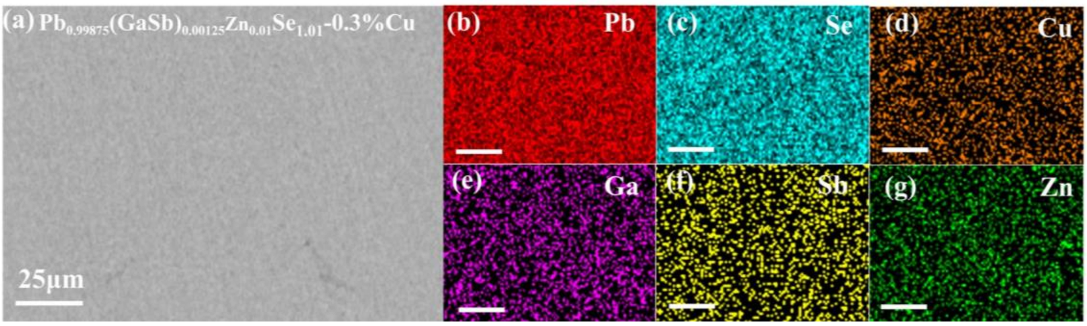

Figure S14. Microstructure and composition analysis of the $Pb_{0.99875}(GaSb)_{0.00125}Zn_{0.01}Se_{1.01}$-0.3%Cu sample. (a) Back-scattered SEM (BSE) image of the specimen. (b-g) EDS mapping of the Figure S14 (a) region.

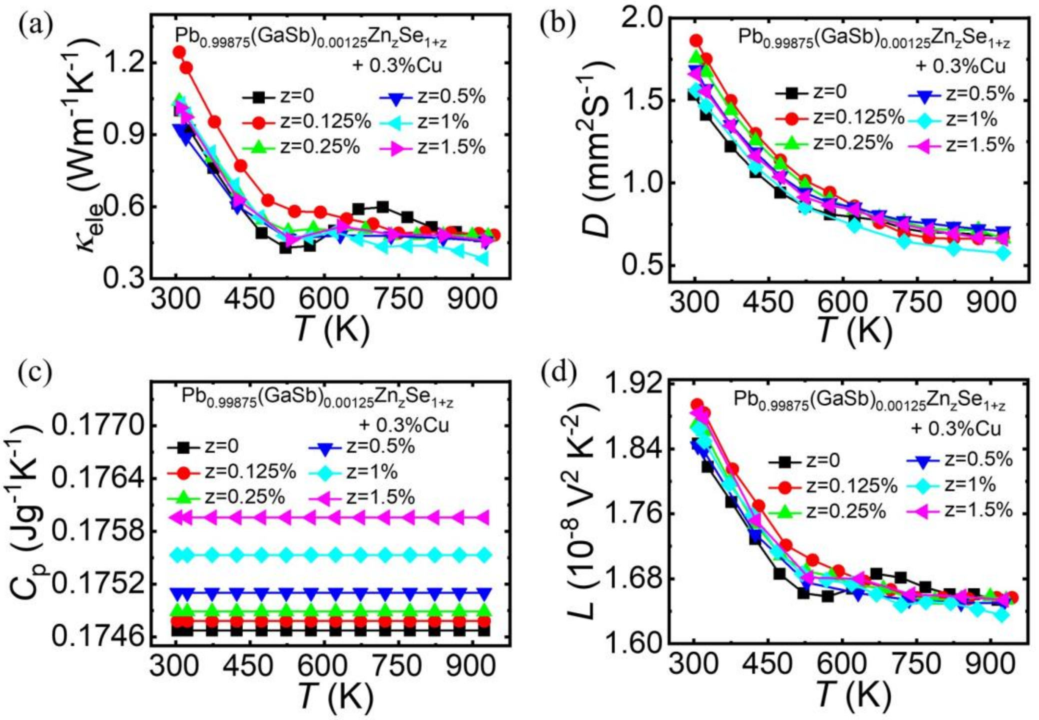

Figure S15. Temperature-dependent (a) electronic thermal conductivity, κ_{ele}; (b) thermal diffusivity, D; (c) heat capacity, C_p; and (d) Lorenz numbers, L for $Pb_{0.99875}(GaSb)_{0.00125}Zn_zSe_{1+z}$-0.3%Cu (z = 0, 0.125%, 0.25%, 0.5%, 1%, and 1.5%) samples.

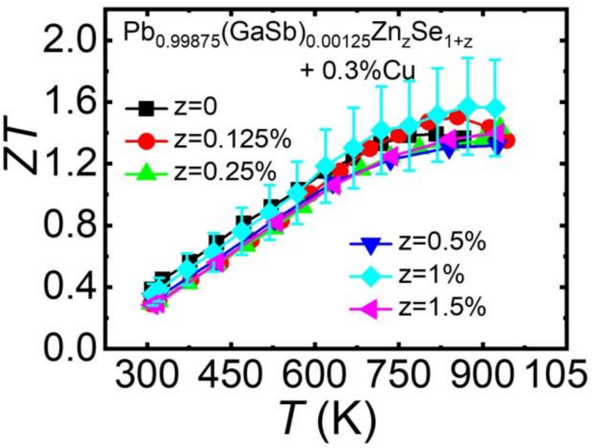

Figure S16. Error bars for ZT as a function of temperature for the $Pb_{0.99875}(GaSb)_{0.00125}Zn_zSe_{1+z}$-0.3%Cu (z = 0, 0.125%, 0.25%, 0.5%, 1%, and 1.5%) samples (The margin of error is 20%).

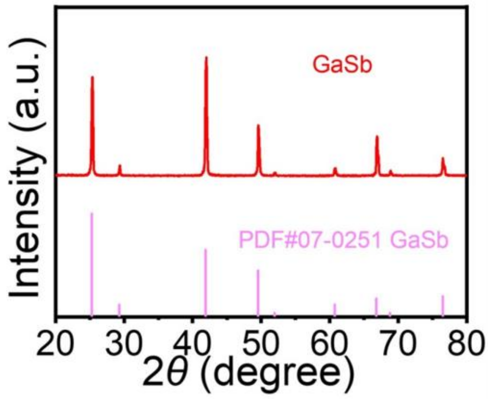

Figure S17. PXRD patterns of GaSb with all peaks indexed by the referenced patterns JCPDS 78−1055.

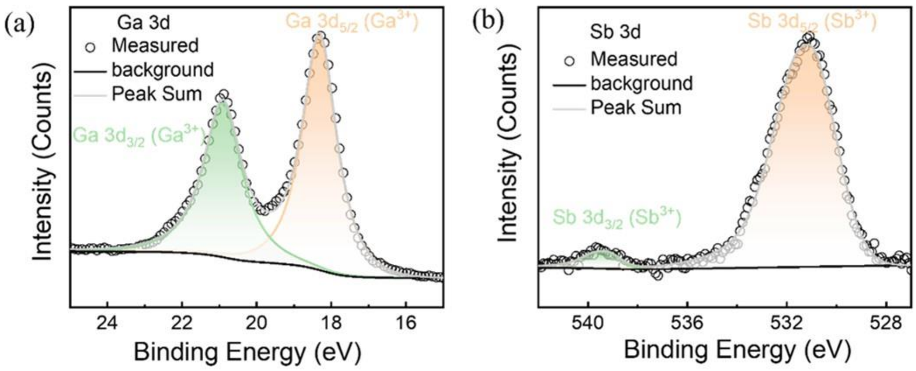

Figure S18. High-resolution XPS spectra of $Pb_{0.99875}(GaSb)_{0.00125}Se$ sample:(a) Ga 3d and (b) Sb 3d.

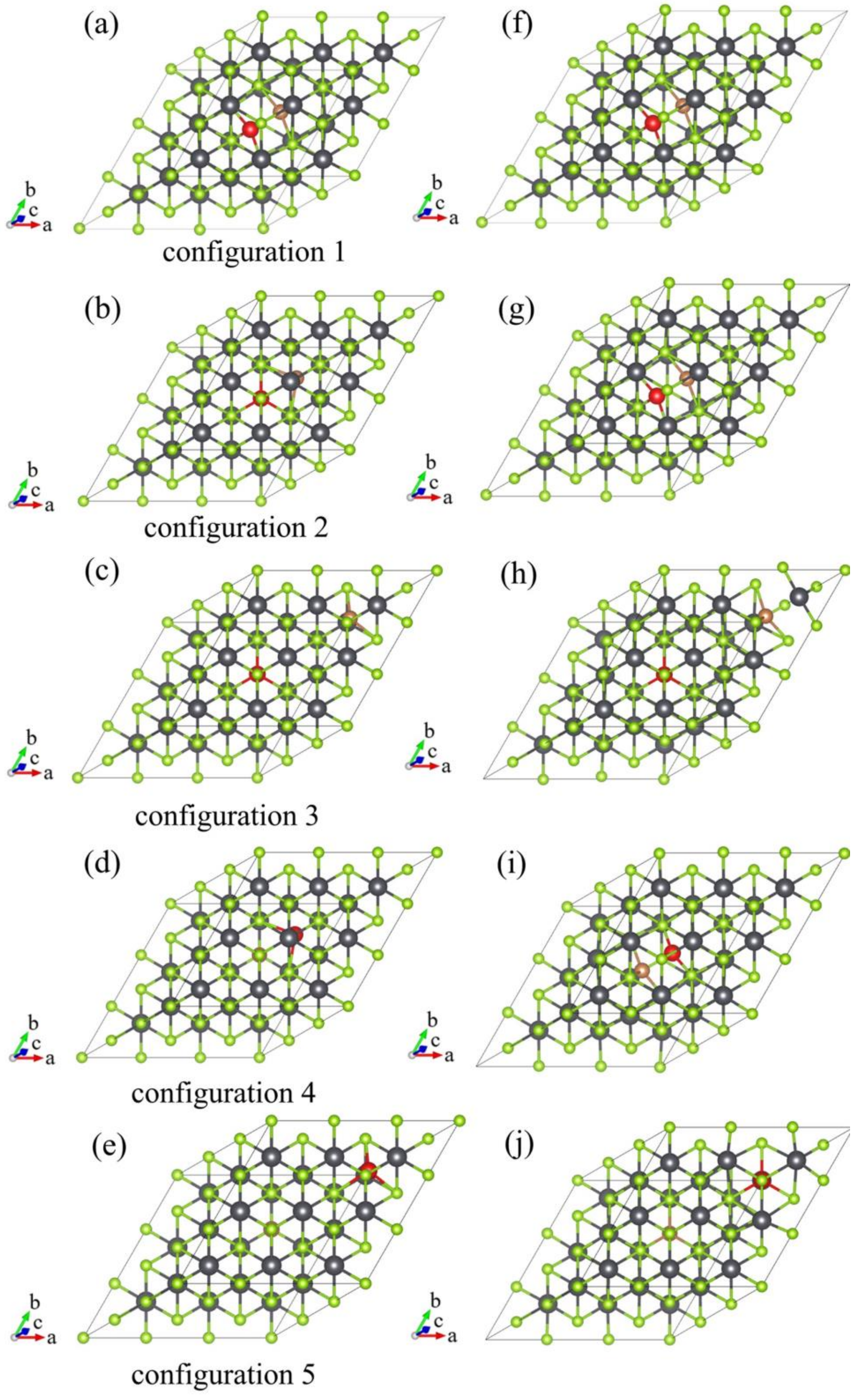

Figure S19. The possible configurations for GaSb-doped PbSe: (a) configuration 1, Ga and Sb substitute for one Pb atom; configuration 2 and configuration 3, Ga substitutes one Pb atom while the Sb atom is positioned in an interstitial site close to (b) or far from (c) the Ga atom; configuration 4 and configuration 5, Sb substitutes one Pb atom while the Ga atom is located in an interstitial site close to (d) or far from (e) the Sb atom and (f-j) are the corresponding optimize configurations of (a-e).

Table S1. The density of the $Pb_{1-x}(GaSb)_xSe$ (x = 0.05%, 0.075%, 0.1%, 0.125%, 0.15%, and 0.175%) samples after SPS.

Sample	Measured density (g cm^{-3})	Relative density (%)
x=0.005%	8.222	99.06
x=0.075%	8.229	99.14
x=0.1%	8.147	98.15
x=0.125%	8.105	97.65
x=0.15%	8.104	97.63
x=0.175%	8.175	98.49

Table S2. The density of the $Pb_{0.99875}(GaSb)_{0.00125}Se$-yCu (y = 0, 0.1%, 0.3%, 0.5%, 0.7%, and 0.9%) samples after SPS.

Sample	Measured density (g cm^{-3})	Relative density (%)
y=0	8.105	97.65
y=0.1%	8.130	97.95
y=0.3%	8.171	98.44
y=0.5%	8.107	97.67
y=0.7%	8.082	97.37
y=0.9%	8.167	98.39

Table S3. The density of the $Pb_{0.99875}(GaSb)_{0.00125}Zn_{0.01}Se_{1.01}$-0.3%Cu (z = 0, 0.125%, 0.25%, 0.5%, 1%, and 1.5%) samples after SPS.

Sample	Measured density (g cm^{-3})	Relative density (%)
z=0	8.171	98.44
z=0.125%	8.137	98.03
z=0.25%	8.191	98.68
z=0.5%	8.212	98.93
z=1%	7.986	96.21
z=1.5%	8.011	96.51

Reference

1. Kamila, H. et al. Analyzing transport properties of p-type Mg_2Si–Mg_2Sn solid solutions: optimization of thermoelectric performance and insight into the electronic band structure. *J. Mater. Chem. A* **7**, 1045-1054 (2019).
